# Transcriptome-Wide m6A Analysis Provides Novel Insights Into Testicular Development and Spermatogenesis in Xia-Nan Cattle

**DOI:** 10.3389/fcell.2021.791221

**Published:** 2021-12-22

**Authors:** Shen-he Liu, Xiao-ya Ma, Ting-ting Yue, Zi-chen Wang, Kun-long Qi, Ji-chao Li, Feng Lin, Hossam E. Rushdi, Yu-yang Gao, Tong Fu, Ming Li, Teng-yun Gao, Li-guo Yang, Xue-lei Han, Ting-xian Deng

**Affiliations:** ^1^ College of Animal Science and Technology, Henan Agricultural University, Zhengzhou, China; ^2^ Guangxi Provincial Key Laboratory of Buffalo Genetics, Breeding and Reproduction Technology, Buffalo Research Institute, Chinese Academy of Agricultural Sciences, Nanning, China; ^3^ Henan Dairy Herd Improvement Co., Ltd, Zhengzhou, China; ^4^ Department of Animal Production, Faculty of Agriculture, Cairo University, Giza, Egypt; ^5^ Henan Dingyuan Cattle Breeding Co., Ltd., Wuhan, China; ^6^ China Ministry of Education, Key Laboratory of Agricultural Animal Genetics, Breeding and Reproduction, College of Animal Science and Technology, Huazhong Agricultural University, Wuhan, China

**Keywords:** cattle, testis, m6A modification, spermatogenesis, semen quality, sequencing

## Abstract

Testis is the primary organ of the male reproductive tract in mammals that plays a substantial role in spermatogenesis. Improvement of our knowledge regarding the molecular mechanisms in testicular development and spermatogenesis will be reflected in producing spermatozoa of superior fertility. Evidence showed that N6-Methyladenosine (m6A) plays a dynamic role in post-transcription gene expression regulation and is strongly associated with production traits. However, the role of m6A in bovine testis has not been investigated yet. In this study, we conducted MeRIP-Seq analysis to explore the expression profiles of the m6A and its potential mechanism underlying spermatogenesis in nine bovine testes at three developmental stages (prepuberty, puberty and postpuberty). The experimental animals with triplicate in each stage were chosen based on their semen volume and sperm motility except for the prepuberty bulls and used for testes collection. By applying MeRIP-Seq analysis, a total of 8,774 m6A peaks and 6,206 m6A genes among the studied groups were identified. All the detected peaks were found to be mainly enriched in the coding region and 3′- untranslated regions. The cross-analysis of m6A and mRNA expression exhibited 502 genes with concomitant changes in the mRNA expression and m6A modification. Notably, 30 candidate genes were located in the largest network of protein-protein interactions. Interestingly, four key node genes (*PLK4, PTEN, EGR1*, and *PSME4*) were associated with the regulation of mammal testis development and spermatogenesis. This study is the first to present a map of RNA m6A modification in bovine testes at distinct ages, and provides new insights into m6A topology and related molecular mechanisms underlying bovine spermatogenesis, and establishes a basis for further studies on spermatogenesis in mammals.

## Introduction

Xia-Nan (XN) cattle, the first specialized beef breed produced by the crossbreeding of French Charolais (male) and Nanyang cattle (female) in China, has important features including fast growth rate and high meat production performance. Enhancing the reproductive performance of XN cattle is an important breeding objective to increase the efficiency and sustainability of this breed as a major beef producer in the Chinese meat market. Testis, as a basic male reproductive organ, plays a critical role in spermatogenesis and steroidogenesis. Bull’s fertility has been always considered a key issue for both cattle breeders and scientists. Different methods and common practices are traditionally used to check male reproductive performance, including a physical assessment of the bull (e.g., volume of the testicles) and semen evaluation (e.g., sperm morphology, concentration, and motility). Although these procedures offered a considerable background on testicular and epididymal function and quantitative production of sperms, infertility due to major reasons was not well recognized. Nowadays, molecular genetics techniques can efficiently target semen quality providing a powerful approach to evaluate the fertility potential of mammalian males (Puglisi et al., 2016; Vendelbo et al., 2021). Establishing an association between spermatogenesis and gene expression profile may enhance a better understanding of the causes of infertility. Improving the testicular function for acceptable production yield of high-quality semen (e.g., healthy spermatozoa) is still the basis for increasing the opportunity for the success rate of obtaining healthy offspring. Spermatogenesis can be divided into three major functional stages including the proliferative stage, meiosis, and maturation stage (Griswold, 2016). Notably, spermatogenesis is strictly regulated by the expression of stage-specific genes at both transcription and post-transcription levels (Ding et al., 2020). Thus, the testes at different developmental stages in XN cattle were taken as the experimental object in the present study, taking into consideration that identifying key regulators and signaling pathways related to testis development and spermatogenesis will provide valuable insights into improving semen quality.

N^6^-methyladenosine (m6A) is the most abundant internal RNA modification that has been recently suggested as a critical post-transcriptional mRNA regulator in most organisms, and is strongly associated with production traits (Shi et al., 2019; Zhang et al., 2020b; Xiong et al., 2021). In mammals, the RNA m6A modification is installed by a methyltransferase complex that mainly includes methyltransferase-like 3 (*METTL3*) and *METTL14*, which are responsible for catalyzing m6A modification (Liu et al., 2014). Wilms’ tumor 1-associated protein (*WTAP*) is another essential member of the core component that interacts with *METTL3* and is required for its localization in the nucleus (Schwartz et al., 2014). RNA m6A modification can be removed by two known demethylases: AlkB homolog 5 (*ALKBH5*) (Zheng et al., 2013) and fat mass and obesity-associated factor (*FTO*) (Jia et al., 2011). RNA m6A modification affects almost all stages of RNA metabolism, like alternative splicing, RNA degradation, nuclear RNA export, and translation (Niu et al., 2013), and these influences can be recognized by a category of proteins primarily composed of the YTH domain family (*YTHDF1-3*) and IGF2BPs (*IGF2BP1-3*) (Dominissini et al., 2012; Fu et al., 2014; Wang et al., 2014). Also, m6A modification can influence the spermatogenic function, for example, the combined deletion of *METTL3* and *METTL14* leads to impaired murine spermiogenesis (Lin et al., 2017). Moreover, m6A demethylase *ALKBH5* deficiency causes compromised spermatogenesis and apoptosis in mouse testis (Zheng et al., 2013). The *YTHDC2* is an m6A-binding protein gene and its knockout is related to infertile in mice (Hsu et al., 2017). Considering the various functions of m6A modification mentioned above in different species, it would seem logical to assume that m6A modification may also affect bovine testis development. During the last few years, continuously incremented works were carried out to investigate the mechanisms of spermatogenesis of cattle by comparative analysis of the gene expression associated with reproductive traits on the molecular level. However, little is known until now about the potential impacts of m6A modification on spermatogenesis in ruminants, in general, and there are no reports that document the association of m6A modification and bovine testis development in XN cattle, in specific.

Therefore, to identify the functional m6A and explore the spermatogenesis mechanism of the testes caused by the m6A RNA modification, we detected the m6A methylomes of bovine testes from birth to adulthood. This enabled us to obtain the transcriptome-wide m6A profiles in bovine testes at different stages and to provide reasonable insights into the roles of m6A modification in the mechanisms underlying spermatogenesis during post-natal testicular development.

## Materials and Methods

### Experiment Design and Sample Collection

The main steps and bioinformatics used for data analysis in the present study is shown in Supplementary Figure S1. A total of sixty-nine clinically healthy XN cattle bulls from Kerchin Cattle Industry (Nanyang) Co., China was randomly selected. These animals were grouped into three groups based on their sexual maturity. The first group indicates the bulls are prepuberty (at birth, n = 23), the second group represents the bulls are puberty (about 1 year old showing heat for the first time, n = 23), and the last group represents the bulls are postpuberty (about 2 years of age, n = 23). In addition, the semen within 1 month of the bulls in puberty and postpuberty was taken by using artificial vagina. Sperm concentration was assessed using a haemocytometer (Bane, 1952). Sperm motility was evaluated based on the methods described (Björndahl et al., 2003). Further, the *t*-test was used to determine the significant difference levels of the semen quality parameters (semen volume, sperm motility and sperm concentration) between the puberty and postpuberty groups. For the puberty and postpuberty groups, the representative individuals of each group were selected in two ways: 1) the semen parameters existed significance differences between them; 2) the semen quality parameters closed to mean value for each group. In the prepuberty, the samples were randomly chosen because the bulls have no semen parameters. Finally, a total of nine bulls (triplicate in each group) were selected based on the above-mentioned criterion. The selected animals were given general anesthesia (Zoletil 50, Virbac Co., France), a combination of zolazepam (5–9 mg/kg, i.m) and tiletamine, and xylazine hydrochloride (1.5–2 mg/kg, i.m) before sampling. Samples of the left testes were collected posterior to castration that performed by professional veterinarians. The length, width, and weight of the left testes were measured. Each testis was divided into three pieces and immediately subjected to snap-freezing in liquid nitrogen and stored at −80°C until RNA extraction.

### RNA Isolation, Library Construction and Sequencing

Total RNA for each testis sample was isolated using the Trizol reagent (Invitrogen, CA, United States) following the manufacturer’s protocols. The quality and quantity of total RNA were determined by Agilent Bioanalyzer 2100 system (Agilent Technologies Inc., CA, United States) and RNA 6000 Nano LabChip Kit (Agilent, Santa Clara, CA, United States) with RIN number >7.0. Poly(A) RNA from total RNA was isolated with Arraystar Seq-Star™ poly(A) mRNA Isolation Kit (Arraystar, MD, United States). The RNA was further fragmented into fragments with an average length of 100 nt using RNA Fragmentation Reagents (Sigma, MO, United States). The fragmented RNA segments were divided into two groups. One group was used to perform m6A RNA immunoprecipitation (IP) using the GenSeq™ m6A RNA IP Kit (GenSeq Inc., China). The other group was utilized to construct the input samples without immunoprecipitation. The IP and input libraries were both constructed with NEBNext^®^ Ultra II Directional RNA Library Prep Kit (New England Biolabs, Inc., MA, United States). The quality of all libraries was measured by the Agilent Bioanalyzer 2100 system (Agilent Technologies Inc., CA, United States). The sequencing of nine cDNA libraries was performed on the Illumina HiseqTM 4000 by Gene Denovo Biotechnology Co., Ltd (Guangzhou, China). The raw data were deposited in the NCBI SRA database (BioProject ID: PRJNA776655).

### Bioinformatics Analysis of m6A-Seq and RNA-Seq Data

TrimGalore v0.6.6 (Krueger, 2021) software was used to eliminate the reads containing adaptor contaminants, low-quality bases, and undetermined bases. Meanwhile, the sequence quality of IP and input of all samples were validated by the FASTP v0.20.1 (Chen et al., 2018) software. Subsequently, the high-quality clean reads were mapped to the *Bos taurus* reference genome (*ARS-UCD1.2*) by HISAT2 ver.2.1.0 (Kim et al., 2015) software with default parameters. Peak calling for mapped reads of IP and input libraries were performed using the exomePeak2 (Meng et al., 2014) package in R. The m6A intensity was visualized using the IGV software (http://www.igv.org/). The identified m6A peaks were carried out to conduct the motif enrichment analysis by MEME (Bailey et al., 2009) and HOMER (Sven et al., 2010) software with default parameters. The ChIPseeker v1.0 (Guangchuang et al., 2015) software was used to annotate the called peaks by intersection with gene architecture. The differential m6A peaks [fold changes (FC) ≥ 2 and adjusted *p*-value < 0.05] between the pairwise comparison groups were analyzed using the exomePeak2 package in R. These differential peaks were annotated using the Ensembl database (*Bos taurus*/*ARS-UCD1.2*). Moreover, the expression level for all mRNAs from input libraries was calculated using StringTie ver. 1.3.5 (Pertea et al., 2016) software. The expression level of each transcript was normalized by the Trimmed Mean of M-values (TMM) implemented in the edgeR R-package. The differential expression analysis for pairwise contrasts was performed using the DESeq2 (Love et al., 2014) package in R. The adjusted *p*-value ≤ 0.05 and FoldChange >1.5 were defined as the cutoff criteria for the differentially expressed mRNAs (DEGs). Gene enrichment analysis was performed by the Gene Ontology (GO) functional analysis and the Kyoto Encyclopedia of Genes and Genomics (KEGG) pathway enrichment using the KEGG Orthology-Based Annotation System (KOBAS) 3.0 with cutoff criteria of *p* ≤ 0.05, aiming to identify their biological significance. The plot results were visualized using the ggplot2 (Wickham, 2016) package in R.

### Quantitative Real-Time PCR Confirmation

Four differentially m6A methylated (DMGs) genes were selected and analyzed by qRT-PCR. Primers were designed using Primer 5.0 software (Supplementary Table S1) and synthesized by Sangon Biotech (Shanghai) Co. Ltd. RevertAid First Strand cDNA Synthesis Kit (Thermo Fisher Scientific, United States) was used to reverse transcribe the total RNAs into cDNA following the manufacturer’s protocols. Then qPCR was conducted using QuantiNova SYBR Green PCR Kit (QIAGEN, Shanghai, China). The *GAPDH* gene was used for normalizing the relative abundance of genes. The 2^−ΔΔCt^ method (Livak and Schmittgen, 2001) was used to analyze the data for all samples in triplicate technical replicates.

### Statistical Analysis

The data are expressed as mean ± standard error of the mean (SEM). The student’s *t*-test and one-way analysis of variance (ANOVA) was performed to determine the significance of the differences between the contrasting groups by using Graphpad Prism 8 software. Differences between means were considered statistically significant when adjusted pairwise comparison between means reached *p*-value ≤ 0.05 (Bonferroni).

## Results

### Testis Source Description

Semen quality parameters of the bulls in puberty and postpuberty, including the semen volume, sperm motility and sperm concentration were analyzed, and their results was listed in Table 1. Our data showed the semen volume and sperm motility in postpuberty were markedly higher (*p* < 0.05), while sperm concentration was not different as compared to bulls in puberty. The Supplementary Figure S2 further showed the detailed information on the semen volume, sperm motility and sperm concentration of bulls in puberty and postpuberty groups. Meanwhile, we selected six animals (three bulls for each group) to subsequently testes collection according to the principle that semen quality parameters closed to mean value. In addition, 3 bulls in prepuberty were randomly chosen.

**TABLE 1:** Evaluations of semen quality and testicular phenotypic parameters of XN bull testes at different ages (mean ± SEM).

Indexes	Prepuberty	Groups^a^	
Semen quality parameters	prepuberty (*n* = 23)	Puberty (*n* = 23)	Postpuberty (*n* = 23)
Semen volume (ml)		4.96 ± 0.26^b^	6.21 ± 0.23^c^
Sperm motility (%)		0.61 ± 0.02^b^	0.67 ± 0.01^c^
Sperm concentration (billion/ml)		1.06 ± 0.07	1.20 ± 0.03
Physical attributed	TY0 (*n* = 3)	TY1 (*n* = 3)	TY2 (*n* = 3)
Weight (g)	4.84 ± 0.89^b^	168.73 ± 5.04^c^	322.81 ± 12.34^d^
Length (Cm)	4.07 ± 0.46^b^	9.97 ± 0.68^c^	13.27 ± 0.44^d^
Width (Cm)	1.70 ± 0.15^b^	5.73 ± 0.09^c^	7.23 ± 0.23^d^

a TY0 = prepuberty; TY1 = puberty; TY2 = postpuberty.

b The different superscript in the same row show significant differences (*p* < 0.05).

c The different superscript in the same row show significant differences (*p* < 0.05).

d The different superscript in the same row show significant differences (*p* < 0.05).

For the above-selected 9 bulls, we measured their weight, length, and width of left testes, and their estimates are listed in Table 1. The results showed that all the tested testicular phenotypic parameters varied significantly (*p* < 0.05) among the groups studied. According to their semen quality and phenotypic difference, 9 testes samples of XN males at three developmental stages (prepuberty = TY0, in puberty = TY1, and postpuberty = TY2) were employed for further m6A-seq analysis. Each stage was in triplicate.

### Transcriptome-Wide m6A-Seq Revealed m6A Modification Patterns During Testis Development

In the present study, the bull testes in prepuberty, puberty and postpuberty were used for m6A-Seq and RNA-Seq assays, with three replicates for each group. For the m6A-Seq, we obtained approximately 4.10 million raw reads for each sample, and about 3.39 million clean reads were mapped to the bovine reference genome for each animal (Supplementary Table S2). Regarding RNA-Seq, approximately 4.20 million raw reads for each individual were generated, and about 4.06 million valid reads were mapped to the reference genome for each individual (Supplementary Table S2). The proportions of mapped reads ranged from 86.39 to 97.12%, correspondingly (Supplementary Table S2). These results demonstrated that the high-quality sequence data obtained are proper to be used in subsequent analysis.

**FIGURE 1:**
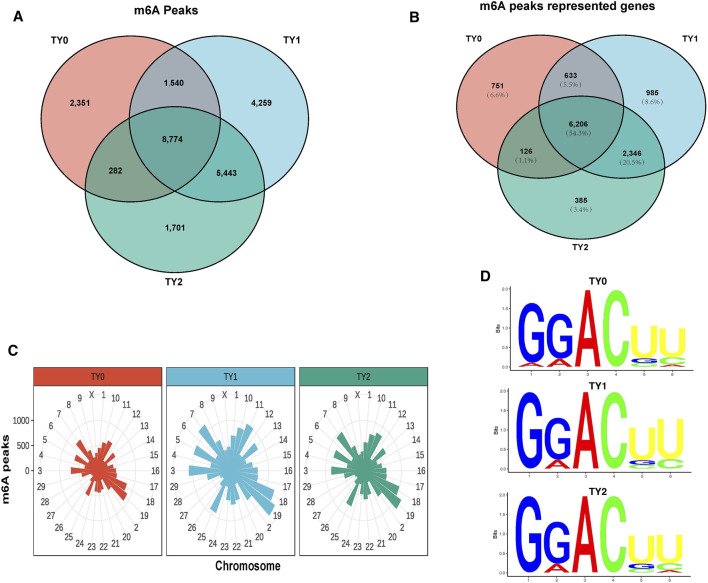
Transcriptome-wide m6A analysis in bovine testes. **(A)** The number of common and specific m6A peaks in three groups. **(B)** The Venn diagram shows the m6A-related transcripts in three groups. **(C)** Distribution of m6A peaks across chromosomes in the three groups. **(D)** The top motifs enriched from m6A peaks were identified among the studied groups.

Using the exomePeak2 analysis, a total of 12,947, 20,016, and 16,200 m6A peaks were detected in the TY0, TY1, and TY2 groups, respectively (Figure 1A). Among them, a total of 2,351, 4,259, and 1,701 specific peaks were observed in the TY0, TY1, and TY2, respectively, reflecting the significant difference among the studied groups in total m6A modification trends. Likewise, a total of 7,716, 10,170, and 9,063 genes were annotated in the TY0, TY1, and TY2 groups, respectively (Figure 1B). In addition, 8,774 peaks were consistently observed in three groups, and 6,206 genes (54.3% of total genes) within the studied groups were modified by m6A.

Considering genome coverage pattern, the m6A distribution analysis revealed that the m6A peaks for each group differentially distributed on bovine chromosomes (Figure 1C). Most of the m6A peaks enriched in chromosome 19 in all studied groups. The motif analysis results indicated that the three studied groups had the classic m6A RRACH consensus sequences (Figure 1D). This enabled us to obtain the high credibility of the m6A peaks and revealed the presence of a prevailing methylated modification mechanism.

### Analysis of m6A Modification Distribution in Testes Transcriptome

**FIGURE 2:**
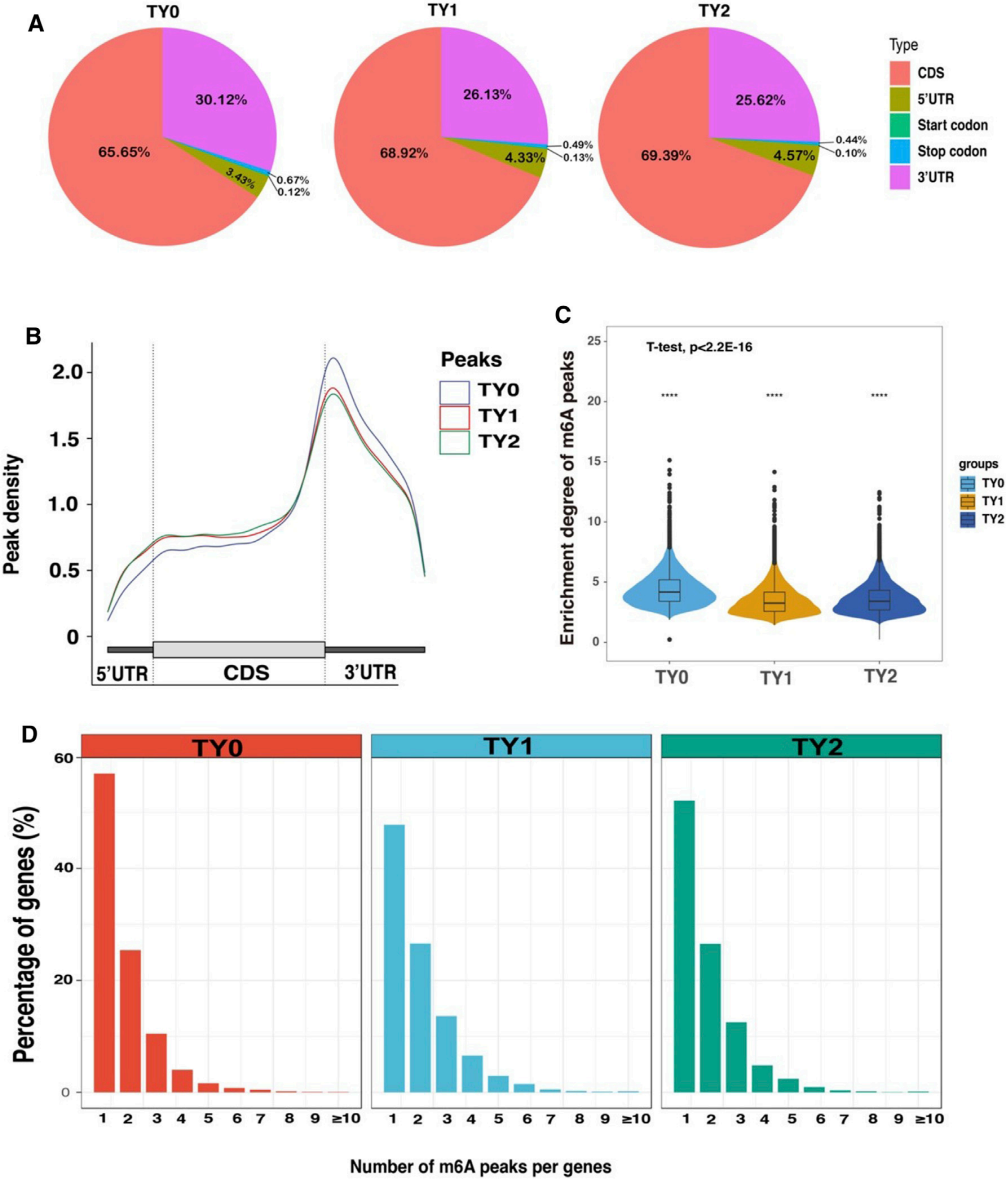
Overview of m6A methylation profiles in bovine testes. **(A)** Pie charts demonstrating m6A peak distribution in the gene structures of mRNAs. **(B)** Metagene plots displaying the regions of m6A peaks identified across the transcripts in TY0, TY1, and TY2 groups. **(C)** Violin plot displays the distribution of enrichment degree of m6A peaks in each group. **(D)** The number of m6A peaks per gene in the TY0, TY1, and TY2 groups.

To investigate the preferential locations of m6A in transcripts, we explored the profiles of m6A peaks in the mRNA transcriptome by coordinating the bovine reference genome. The transcript was divided into the following regions: the 5′untranslated regions (5′UTRs), near the start codon, CDS, near the stop codon, and the 3′untranslated regions (3′UTRs). The analysis revealed that enrichment of m6A modified peaks was highest in the CDS followed by the 3′UTRs, 5′UTRs, stop codon region, and start codon region in the studied groups (Figure 2A). The distribution of m6A peak density in each group exhibited similar trends (Figure 2B). In addition, we observed that the number of m6A modified peaks enriched in the CDS region was higher in the both puberty and postpuberty groups than in the prepuberty group, but the opposite trend was observed for enrichment near the stop codon. Thereafter, the enrichment degree of m6A peaks significantly varied among all the pairwise groups (*p*-value < 2.21E-16; Figure 2C). The distribution of m6A modified peaks with each gene was explored and it showed that almost >50% of affected genes hold only one m6A peak and the majority of genes harbored one to three m6A peaks (Figure 2D).

### Differentially Methylated Gene Analysis

**FIGURE 3:**
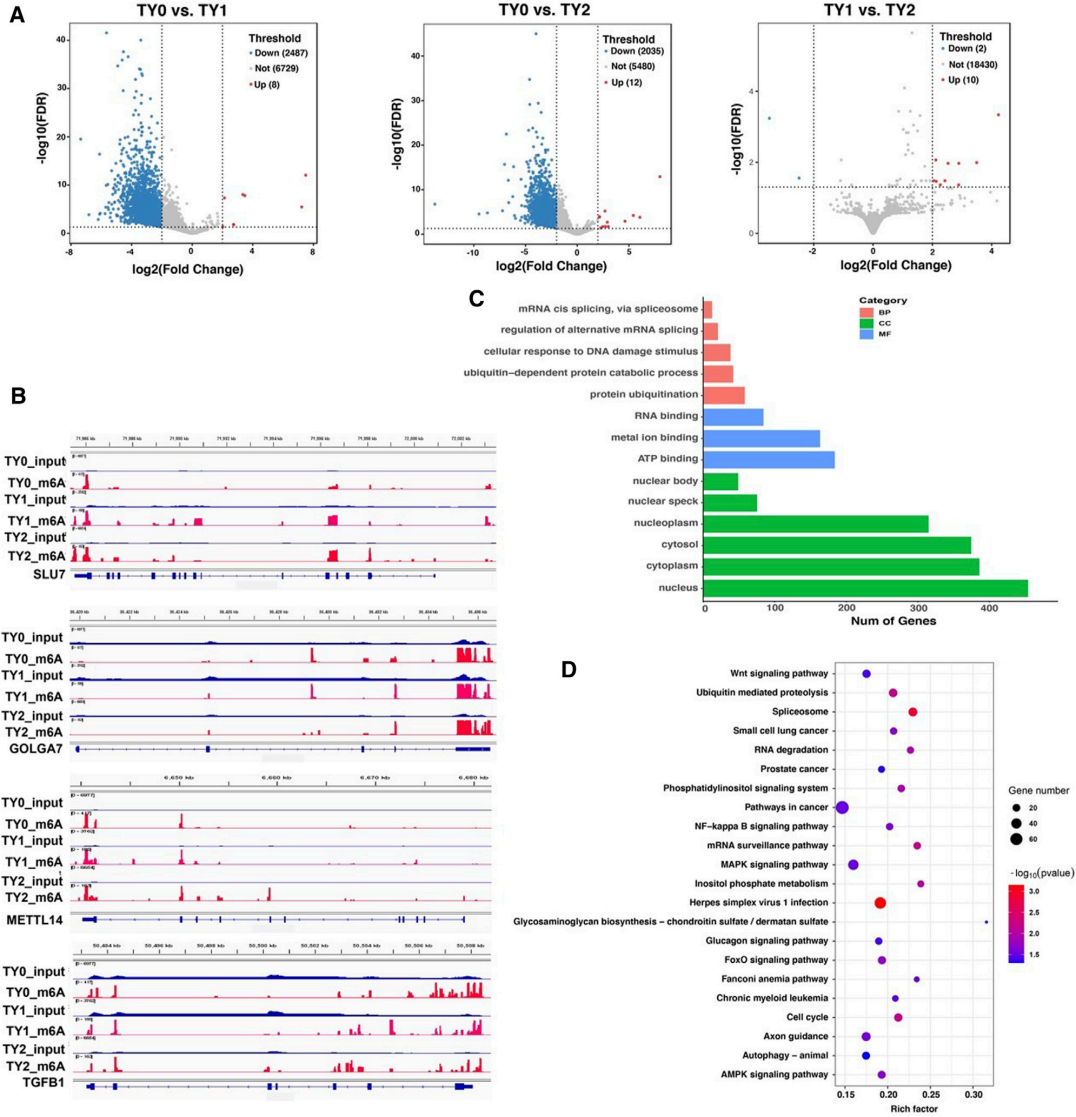
Distribution of significantly differential m6A peaks between the pairwise comparison groups. **(A)** Volcano plots showing the differential peaks between the studied groups. **(B)** Data visualization analysis of differential m6A peaks in the selected mRNAs (*SLU7*, *GOLGA7*, *METTL14*, and *TGFB1*) among the studied groups. **(C)** GO analysis for differentially methylated genes. **(D)** KEGG enrichment analysis for differentially methylated genes.

To explore the potential function of the m6A modification in bovine testes, a pairwise comparison was applied to scan the DMGs. Compared to the TY0 group, 2,495 significantly differential m6A peaks within 2,036 mRNAs were found in the TY1 group, while 2,047 differential peaks within 1,719 mRNAs were detected in the TY2 group (Figure 3A; Supplementary Table S3). In addition, we found 22 differential m6A peaks within 11 mRNAs in the TY2 group compared to the TY1 group. Several randomly selected mRNAs, including *SLU7*, *GOLGA7*, *METTL14*, and *TGFB1*, showed significantly hypermethylated peaks as presented in Figure 3B.

Moreover, the GO and KEGG enrichment analyses for all DMGs were performed to demonstrate the important function of m6A modification in bovine testes. GO analysis revealed that all the DMGs were mainly annotated into the nucleus, cytoplasm (ontology: cellular component), metal ion binding, RNA binding and ATP binding (ontology: molecular function), and protein ubiquitination, ubiquitin-dependent protein catabolic process (ontology: biological process) (Figure 3C). KEGG enrichment analysis demonstrated that the DMGs were significantly implicated with the MAPK signaling pathway, Herpes simplex virus 1 infection, Axon guidance, and FoxO signaling pathway, besides other methylated genes (Figure 3D).

### RNA-Seq Identification of Differentially Expressed Genes

**FIGURE 4:**
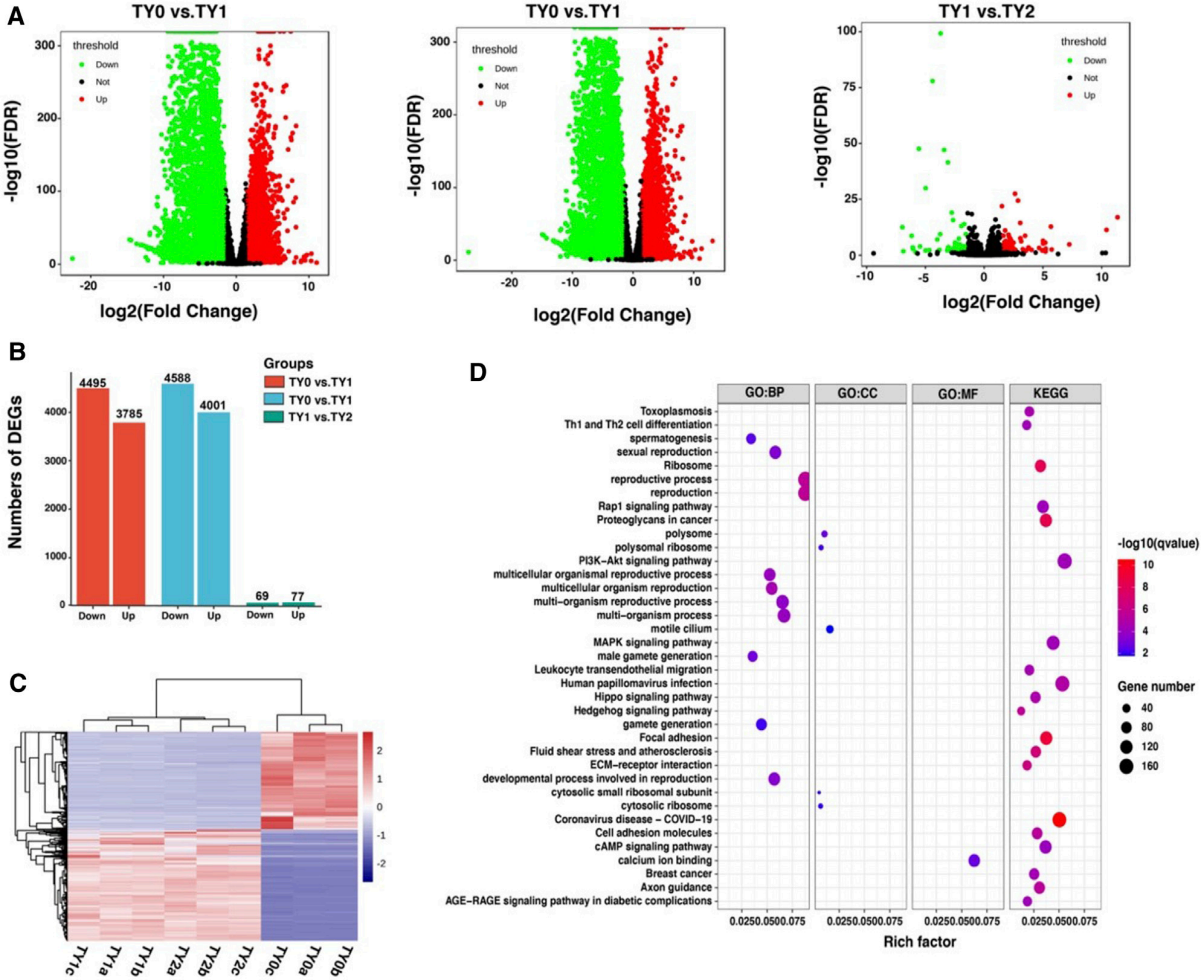
Differential expression analysis of RNA-Seq data among the studied groups. **(A)** Volcano plots showing the differential expressed genes between the studied groups. **(B)** Barplot showing the number of up-and down-regulated DEGs. **(C)** Heatmap plot of all DEGs among the studied groups. **(D)** GO and KEGG enrichment analysis of all DEGs.

Using the RNA-Seq technique, a total of 8,280, 8,589, and 146 DEGs were detected between TY0 *vs.* TY1, TY0 *vs.* TY2, and TY1 *vs.* TY2, respectively (Figure 4A). Correspondingly, a set of 3,785, 4,001, and 77 up-regulated as well as 4,495, 4,588, and 69 down-regulated were found, respectively (Figure 4B). The hierarchical cluster expression pattern of the DEGs was shown in Figure 4C. Furthermore, all the DEGs were mainly enriched to 17 GO terms and 20 KEGG pathways (Figure 4D). Notably, most DEGs were highly annotated into the reproduction, reproductive process, multicellular organism reproduction, developmental process involved in reproduction, sexual reproduction, spermatogenesis, male gamete generation (ontology: biological process) and motile cilium (ontology: cellular component) and calcium ion binding (ontology: molecular function) of GO biological process. In addition, the PI3K-Akt signaling pathway is the most significant enrichment pathway for all DEGs identified, followed by MAPK signaling pathway and cAMP signaling pathway etc.

### Conjoint Analysis of m6A-Seq and RNA-Seq Data

**FIGURE 5:**
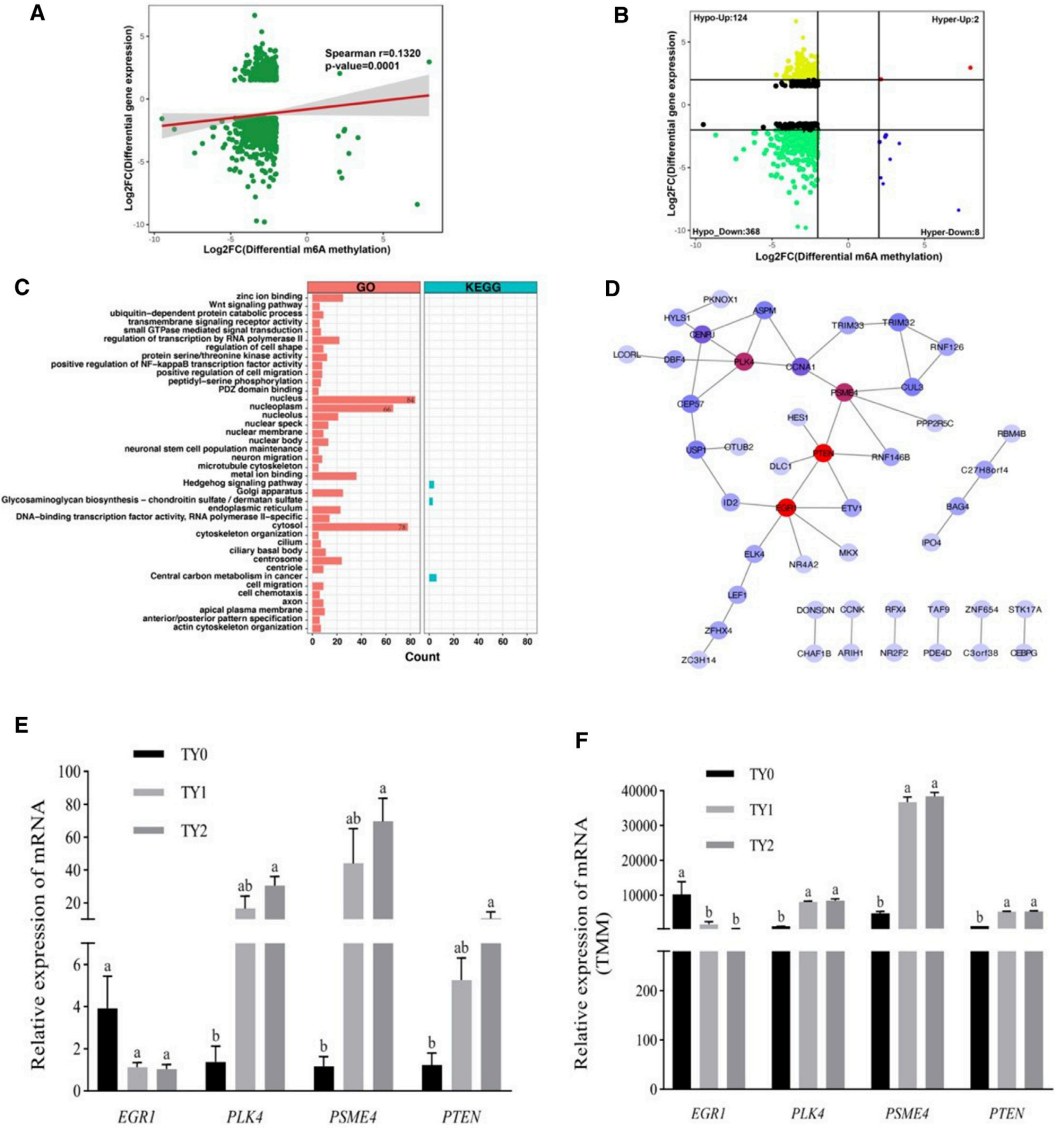
Conjoint analysis of m6A-Seq and RNA-Seq data. **(A)** Dot plot of Log2 FC (mRNA expression) versus Log2 FC (differential m6A methylation) revealing a positive association between total m6A methylation and level of mRNA expression. **(B)** Four quadrant plots showing differentially expressed genes with differentially methylated m6A peaks. **(C)** GO and KEGG pathway enrichment analysis of the genes with a significant change in both m6A and mRNA levels. **(D)** Protein-protein interactions (PPIs) of the genes enriched in the nucleus of GO terms. The red circle represents the node with a high degree, while the blue circle indicates the node with a low degree. **(E)** The relative mRNA levels were determined by the qPCR of four hub genes in three groups. **(F)** The genes change levels based on RNA-Seq data.

To explore the potential relationship between m6A modification and gene expression, we performed a cross-analysis of the m6A-seq and RNA-seq data. As shown in Figure 5A, a positive correlation between differentially methylated peaks and gene expression levels is obvious (*p* = 0.0001, Spearman r = 0.1320). Based on the principle that absolute value of both X and Y axes was greater than 2, the results of the four-quadrant diagram analysis revealed that there were 502 differentially methylated genes, of which 124 genes belonged to the hypo-up, 2 genes to the hyper-up, 368 genes to the hypo-down, and 8 genes to the hyper-down quadrants (Figure 5B). Further, all of these genes were utilized for GO and KEGG pathway analyses (Figure 5C). Most of them were significantly enriched for 37 GO terms and 3 KEGG pathways. Interestingly, 84 genes were annotated into the nucleus of the GO term. The STRING analysis revealed that these genes were clustered into 8 PPIs networks (Figure 5D). Of them, 30 candidate genes were located in the largest PPIs network (Table 2). Further, 4 hub DMGs (*PLK4, PTEN, EGR1*, and *PSME4*) were selected and analyzed by RT-qPCR. The results of qPCR showed that the expression level of the 4 hub DMGs displayed a similar tendency with that of the RNA-Seq (Figures 5E,F). The above results suggested that these candidate genes may have crucial roles in spermatogenesis during bovine testis development.

**TABLE 2:** List of 30 genes with significant changes in m6A and mRNA transcript abundance in bovine testes.

Gene	Symbol	Pattern	m6A level change					mRNA level change	
			Chr	Peak start	Peak end	diff.FC	diff.p	log2(FC)	Qvalue
ENSBTAG00000007860	ASPM	Hypo_Down	16	76,041,095	76,041,295	−2.340	0.010	−3.237	0.000
ENSBTAG00000007284	CCNA1	Hypo_Down	12	25,110,778	25,110,878	−2.405	0.000	−5.692	0.000
ENSBTAG00000017048	CENPJ	Hypo_Down	12	36,496,130	36,496,355	−4.685	0.000	−2.497	0.000
ENSBTAG00000016547	CEP57	Hypo_Down	15	14,448,587	14,448,637	−4.879	0.000	−3.235	0.000
ENSBTAG00000021769	CUL3	Hypo_Down	2	112,587,000	112,587,325	−3.625	0.000	−3.316	0.000
ENSBTAG00000014711	DBF4	Hypo_Down	4	32,288,748	32,288,948	−2.824	0.007	−3.584	0.000
ENSBTAG00000015541	DLC1	Hypo-Up	27	23,915,744	23,915,994	−2.733	0.000	2.021	0.000
ENSBTAG00000010069	EGR1	Hypo-Up	7	49,829,976	49,830,126	−2.566	0.007	2.527	0.000
ENSBTAG00000004953	ELK4	Hypo_Down	16	3,375,597	3,375,847	−2.808	0.000	−2.929	0.000
ENSBTAG00000015981	ETV1	Hypo_Down	4	22,100,027	22,100,077	−2.966	0.011	−2.703	0.000
ENSBTAG00000000569	HES1	Hypo-Up	1	73,362,983	73,363,058	−2.642	0.000	2.518	0.000
ENSBTAG00000010859	HYLS1	Hypo_Down	29	29,255,406	29,255,531	−3.612	0.000	−2.760	0.000
ENSBTAG00000021187	ID2	Hypo-Up	11	88,605,254	88,605,404	−2.933	0.005	2.841	0.000
ENSBTAG00000046561	LCORL	Hypo_Down	6	37,403,775	37,403,825	−6.199	0.001	−2.257	0.000
ENSBTAG00000006844	LEF1	Hyper-Down	6	17,083,863	17,084,113	3.340	0.000	−3.079	0.000
ENSBTAG00000007678	MKX	Hypo_Down	13	36,892,590	36,892,815	−2.628	0.017	−4.774	0.000
ENSBTAG00000003650	NR4A2	Hypo_Down	2	39,905,150	39,905,250	−3.755	0.001	−2.485	0.000
ENSBTAG00000014981	OTUB2	Hypo_Down	21	58,656,844	58,657,069	−4.620	0.000	−4.883	0.000
ENSBTAG00000014153	PKNOX1	Hypo_Down	1	143,202,058	143,202,108	−2.699	0.000	−2.689	0.000
ENSBTAG00000039552	PLK4	Hypo_Down	17	29,786,990	29,787,090	−6.151	0.000	−3.003	0.000
ENSBTAG00000020192	PPP2R5C	Hypo_Down	21	66,815,607	66,815,782	−2.345	0.000	−3.238	0.000
ENSBTAG00000020262	PSME4	Hypo_Down	11	36,573,366	36,573,416	−2.082	0.028	−3.020	0.000
ENSBTAG00000009498	PTEN	Hypo_Down	26	9,564,008	9,564,180	−3.981	0.000	−2.243	0.000
ENSBTAG00000014349	RNF126	Hypo_Down	7	43,262,899	43,262,949	−2.777	0.000	−2.273	0.000
ENSBTAG00000034531	RNF146B	Hypo_Down	9	24,002,245	24,002,445	−2.178	0.000	−2.044	0.000
ENSBTAG00000017155	TRIM32	Hypo_Down	8	105,886,129	105,886,329	−2.293	0.000	−2.669	0.000
ENSBTAG00000001499	TRIM33	Hypo_Down	3	28,977,136	28,977,286	−2.836	0.000	−2.356	0.000
ENSBTAG00000020451	USP1	Hypo_Down	3	83,118,601	83,118,801	−2.842	0.000	−2.755	0.000
ENSBTAG00000030453	ZC3H14	Hypo_Down	10	100,267,705	100,267,930	−3.124	0.000	−2.682	0.000
ENSBTAG00000033268	ZFHX4	Hypo_Down	14	40,040,613	40,040,713	−2.826	0.012	−2.833	0.000

Chr indicates the chromosome of cattle; diff.FC represents the differential log2 fold change estimates; diff.p indicates the differential *p*-values; FC represents Fold changes. All the data in diff.p and qvalue part are rounded.

## Discussion

Improvement of our knowledge regarding the testis functions developments is vital for gaining our understating regard to the spermatogenesis process. The role of m6A modification influences the mechanisms underlying testis development and spermatogenesis. To our best knowledge, this work is the first comprehensive high-throughput study of RNA methylation in the testes of XN young calves and mature bulls. To explore the potential function of the m6A modification affecting spermatogenesis, we specified three age points (TY0, TY1, and TY2) with significantly different physiological statuses in bovine testis development to analyze the transcriptome-wide m6A profile. The generated data in the present study displayed that a diverse pattern of mRNA methylation have occurred over the testis development. These mRNA m6A sites were mainly concentrated around the CDS and 3′UTRs region (95.28%), in agreement with the distributional characteristics of the mammalian transcriptomes (Wen et al., 2020). Our data showed that although the m6A peaks for each group markedly varied on different chromosomes, they had a highest expressed distribution in chromosome 19 within groups. The great differences in the genome coverage of reads among chromosomes observed in the present study may be attributed to the variation in the gene density of the bovine chromosomes. In addition, these m6A sites tended to occur in the conserved motif sequence “RRACH”, similar to that reported in other animals, such as the goat (Wang et al., 2020), sheep (Lu et al., 2019), and yak (Zhang et al., 2020a). This finding supported our hypothesis on the presence of a predominant methylated modification mechanism based on the m6A peaks identified.

In the study, a total of 2,278 unique DMGs were identified among all the pairwise groups. Differential m6A methylation has proved to be responsible for tissue or organ differentiation and development (Wen et al., 2020). Regarding the function and pathway of DMGs, we performed GO and KEGG enrichment analysis for DMGs. Importantly, GO analysis revealed that all the DMGs were mainly annotated into the nucleus, cytoplasm, metal ion binding, RNA binding, ATP binding, protein ubiquitination, and ubiquitin-dependent protein catabolic process. Earlier reports showed that metal ion binding was involved in porcine spermatogenesis (Luo et al., 2015) and horse testis development (Han et al., 2020). A growing body of evidence supports the fact that protein ubiquitination also has a critical role in the regulation of spermatogenesis in testes of the mouse (Guo et al., 2021) and buffalo (Huang et al., 2020; Zhang et al., 2021). Similarly, RNA binding was also essential for spermatogenesis (Venables and Eperon, 1999; Paronetto and Sette, 2010; Jan et al., 2017). Moreover, KEGG enrichment analysis revealed that the DMGs were significantly implicated in the MAPK signaling pathway, Herpes simplex virus 1 infection, Axon guidance, and FoxO signaling pathway. Ni et al. (2019) found that the MAPK signaling pathway regulates dynamics of tight junctions and adherents junctions, proliferation, and meiosis of germ cells, proliferation and lactate production of Sertoli cells. Accumulating evidence has demonstrated that the FoxO signaling pathway was involved in the regulation of spermatogenesis process (Huang et al., 2016b; Ge et al., 2019; Hu et al., 2021). Therefore, above GO terms and pathways results suggested that the DMGss identified were related to bovine testis development and spermatogenesis process.

Likewise, our results showed that there are 8,952 unique DEGs were observed among all the pairwise groups. We observed that most DEGs were highly annotated into the reproduction, reproductive process, multicellular organism reproduction, developmental process involved in reproduction, sexual reproduction, spermatogenesis, male gamete generation (ontology: biological process) of the GO biological process, indicating that the identified DEGs in the present study were reliable. Notably, the PI3K-Akt signaling pathway was found to be the most significant enrichment pathway for all DEGs. Many studies have demonstrated that the PI3K-Akt signaling pathway was involved in the regulation of spermatogenesis (Duan et al., 2016; Deng et al., 2020; Ni et al., 2020). In brief, our findings suggested that the DEGs were strongly associated with the testis development and spermatogenesis.

A surprising finding was that the enrichment degree of m6A peaks (Figure 2C), intensity of the DMGs (Figure 4A) and DEGs (Figure 5A) exhibited marked differences between before (TY0) and after puberty (TY1 and TY2 groups), suggesting that m6A modification and their gene expression mainly involved in the bovine testicular development and spermatogenesis. To further explore the potential genes underlying bovine testis development and spermatogenesis, the integrated analysis of m6A-Seq and mRNA-Seq data were performed. Results revealed that a positive correlation existed in differentially methylated peaks and gene expression levels, and 502 genes with concomitant changes in the mRNA expression and m6A modification. Interestingly, 84 genes were annotated into the nucleus of GO term, of which 30 candidate genes were located in the largest PPIs network. Importantly, four key node genes (*PLK4, PTEN, EGR1*, and *PSME4*) were observed on the largest PPIs network. Notably, the results of qPCR also supported that the expression level of the 4 selected DMGs exhibited marked differences between before and after puberty, which displayed a similar tendency with that of the RNA-Seq. These results indicated the putative role of these four genes in bovine testis development and spermatogenesis. It was noted that the four genes have already been reported to regulate the mammalian testis development and spermatogenesis. For example, the *PLK4* gene was highly expressed in testes during both pre- and post-natal stages and had a role in the initiation of spermatogenesis (Harris et al., 2011). Moreover, Miyamoto et al. (2016) reported that a mutation in *PLK4* causing azoospermia in a man with Sertoli cell-only syndrome. Dorostghoal et al. (2020) found that sperm *miR-26a-5p* and its target *PTEN* transcript content may contribute to the etiology of male infertility in unexplained infertile patients. More specifically, Neirijnck et al. (2019) reported that ablation of the *PTEN* appears dispensable for Sertoli cell proliferation and spermatogenesis, where inactivation of *PTEN* gene in the absence of Insr and Igf1r rescued the Sertoli cell proliferation rate during late fetal development, testis size, and sperm production. Besides, the *EGR1* gene has a role in maintaining spermatogonia stem cells’ self-renewal and is a target to better understand the molecular basis of spermatogenesis (Sisakhtnezhad and Heshmati, 2018). Also, the *EGR1* gene can act as an activator of the sex-determining region Y box 18 promoter (Petrovic et al., 2010)*.* Additionally, the loss of *PSME4* gene led to a marked reduction in male fertility, due to defects in spermatogenesis observed in meiotic spermatocytes and also during the maturation of postmeiotic haploid spermatids (Khor et al., 2006). Huang et al. (2016a) reported that the double knockout of *PSME3* and *PSME4* genes in mice resulted in completely infertile males. These findings suggested that m6A modifications play an essential role during bovine testis development and spermatogenesis.

## Conclusion

We identified 8,774 m6A peaks and 6,206 m6A genes among the studied groups by using MeRIP-Seq analysis. The cross-analysis of m6A and mRNA expression revealed 502 genes with concomitant changes in the mRNA expression and m6A modification. Further, 30 candidate genes were found to be located in the largest network of protein-protein interactions. Notably, four key node genes (*PLK4, PTEN, EGR1*, and *PSME4*) have been implicated in the regulation of mammalian testis development and spermatogenesis. The present study is pioneer to present a map of RNA m6A modification in bovine testes at different ages, and provides novel insights into m6A topology and associated molecular mechanisms underlying bovine spermatogenesis, and provides a basis for future research on mammalian testis development and spermatogenesis.

## Data Availability

The datasets presented in this study can be found in online repositories. The names of the repository/repositories and accession number(s) can be found below: NCBI SRA; PRJNA776655.
