# Supplementary figures and images for: Transcriptome-Wide m6A Analysis Provides Novel Insights Into Testicular Development and Spermatogenesis in Xia-Nan Cattle
Source: Front Cell Dev Biol. 2021 Dec 22;9:791221. doi: 10.3389/fcell.2021.791221 (PMC8728086; doi:10.3389/fcell.2021.791221)

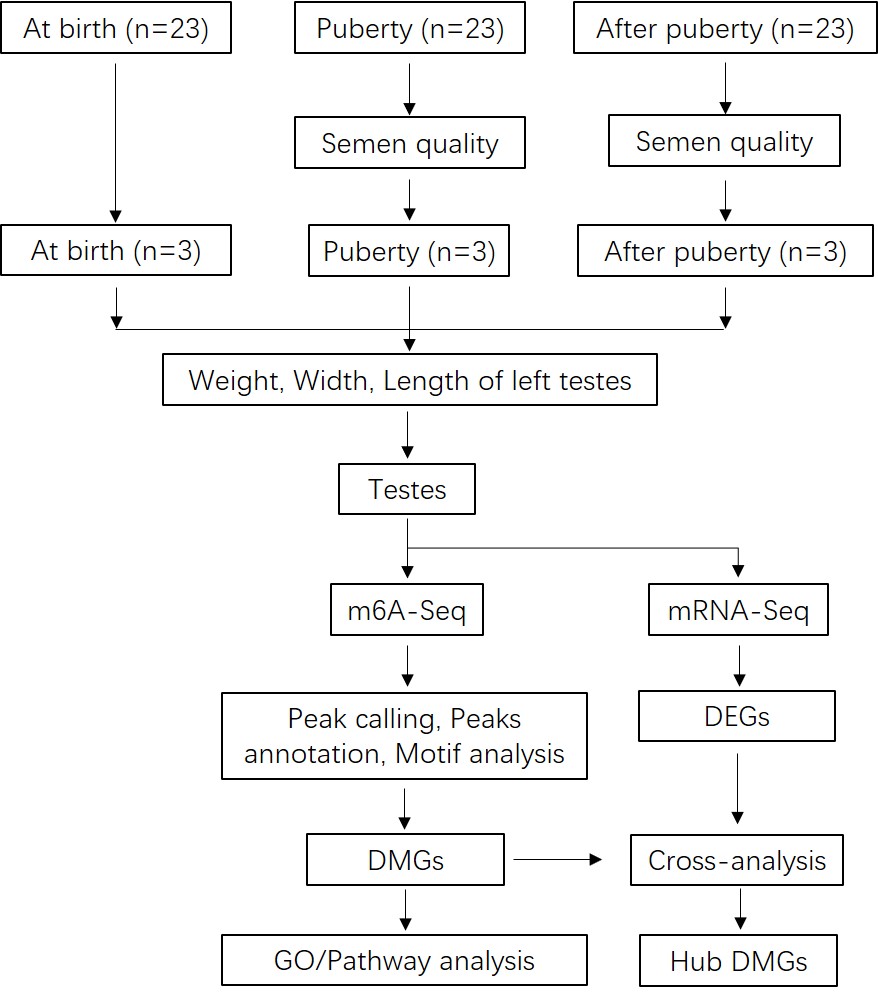

Supplement: Supplementary file 3 [file Image1.JPEG]
